# Production of xylanase by *Aspergillus niger* GIO and *Bacillus megaterium* through solid-state fermentation

**DOI:** 10.1099/acmi.0.000506.v5

**Published:** 2023-06-14

**Authors:** Samuel Adedayo Fasiku, Mobolaji Akeem Bello, Olubusola Ayoola Odeniyi

**Affiliations:** ^1^​ Department of Biological Sciences, Ajayi Crowther University, Oyo Town, Oyo State, Nigeria; ^2^​ Department of Microbiology, University of Ibadan, Ibadan, Nigeria

**Keywords:** xylanase, alkaline-pretreated maize straw, *Aspergillus niger*, *Bacillus megaterium*, solid-state fermentation

## Abstract

Xylanase breaks xylan down to xylose, which is used in industries such as pulp and paper, food and feed, among others. The utilization of wastes for xylanase production is economical, hence this work aimed at producing xylanase through solid-state fermentation and characterizing the enzyme. Xylanase-producing strains of *Bacillus megaterium* and *Aspergillus niger* GIO were inoculated separately in a 5 and 10 day solid fermentation study on maize straw, rice straw, sawdust, corn cob, sugarcane bagasse, conifer litters, alkaline-pretreated maize straw (APM) and combined alkaline and biological-pretreated maize straw, respectively. The best substrate was selected for xylanase production. The crude enzyme was extracted from the fermentation medium and xylanase activity was characterized using parameters such as temperature, cations, pH and surfactants. Among different substrates, the highest xylanase activity of 3.18 U ml^−1^ was recorded when *A. niger* GIO was grown on APM. The xylanase produced by *A. niger* GIO and *B. megaterium* had the highest activities (3.67 U ml^−1^ and 3.36 U ml^−1^) at 40 °C after 30 and 45 min of incubation, respectively. Optimal xylanase activities (4.58 and 3.58 U ml^−1^) of *A. niger* GIO and *B. megaterium *, respectively, were observed at pH 5.0 and 6.2. All cations used enhanced xylanase activities except magnesium ion. Sodium dodecyl sulfate supported the highest xylanase activity of 6.13 and 6.90 U ml^−1^ for *A. niger* GIO and *B. megaterium *, respectively. High yields of xylanase were obtained from *A. niger* GIO and *B. megaterium* cultivated on APM. The xylanase activities were affected by pH, temperature, surfactants and cations.

## Data Summary

No extra data were generated apart from those presented in the Results section.

## Introduction

Lignocellulosic materials are abundant in nature and are principally made up of cellulose, hemicellulose and lignin [1, 2]. Hemicellulose is the second most plentiful biological polymer in the world after cellulose and the main component of hemicellulose is xylan, which is broken down to xylose by the enzyme called xylanase [3]. Xylanase is a hydrolytic enzyme that breaks the β−1, 4-glycosidic bond of xylan in lignocellulolytic substrates to release xylose. Non-production of toxic materials is one of the advantages of using enzyme (xylanase) to break down xylan to xylose when compared with other methods of breaking down xylan [4–6]

Xylanases have been applied in juice clarification [7], desizing yarn before weaving, deinking, biobleaching, improving the rheological property of bread, removal of waxy material from plant fibre, biorefinery [8], enhancement of plant immunity [9] and resistance to pathogens [9]. The use of xylan as a substrate for the production of xylanase has been discouraged because of the high cost of xylan [10]. Thus there is a need for alternative substrates that will contain xylan at a low cost. Lignocellulosic substrates such as agro-wastes can be used as alternatives to xylan in the production of xylanase because these substrates contain xylan and other nutrients necessary for the growth of micro-organisms [10, 11]. Many micro-organisms such as *Bacillus* spp. [3, 7, 12, 13], *Aspergillus* spp. [14–16], *Penicillium* spp. [10, 17], *Fusarium* spp. [11, 18], *Streptomyces* spp. [19, 20], *Trichoderma* spp. [21, 22] and others have been used to produce xylanase. Many parameters, such as temperature, pH, metal ions, detergents and others, affect the activities of xylanase [3, 16].

Maize straw, conifer litters, rice straw, sugarcane bagasse, sawdust, corn cob and other agro residues are wastes in the environment causing pollution. Utilization of these wastes to produce xylanase, an enzyme with many applications, will create safer environments and result in the production of a value-added product (xylanase). This work aimed at producing xylanase using *Aspergillus niger* GIO and *Bacillus megaterium* with agro-wastes, and characterization of the produced enzyme.

## Methods

### Screening for xylanase

*A. niger* GIO (accession number: MZ747463) and *B. megaterium* (characterized and identified according to Sneath *et al*. [23]) used in this work were collected from the Department of Biological Sciences, Ajayi Crowther University, Oyo-Town, Nigeria. Potato dextrose agar and nutrient agar supplemented separately with 0.5 % beechwood xylan (Sigma-Aldrich, Germany) were sterilized at 121 ℃ for 15 min. *A. niger* GIO and *B. megaterium* were inoculated into the supplemented potato dextrose agar and nutrient agar, respectively. *B. megaterium* was incubated at 37 ℃ for 2 days, while *A. niger* GIO was incubated at 28±2 ℃ for 5 days. The incubated plates were afterwards flooded with Lugol’s iodine for 5 min, after which the stain was poured off and a clear zone around the point of inoculation against the dark colour of undegraded xylan indicated that the organism produced xylanase [24].

### Production of xylanase

The ability of the micro-organisms to utilize the following as substrates for the production of xylanase was investigated: maize straw, corn cobs, sugarcane bagasse, conifer litters, sawdust, rice straw, alkaline-pretreated maize straw (APM) and combined (alkanline and biological) pretreated maize straw. Each substrate (5 g) was weighed into separate polythene bags and mixed with 15 ml of a mineral basal solution containing the following in g l^−1^ – glucose 10.0; (NH_4_)_2_SO_4_ 1.0; MgSO_4_·7H_2_O 0.5; KCl 0.5; FeSO_4_ 0.01; MnSO_4_ 0.01. They were sterilized at 121 ℃ for 15 min and allowed to cool. One millilitre of 0.5 MacFarland standard of *B. megaterium* was inoculated into each of the mixtures while four circular plugs (7 mm diameter) of *A. niger* GIO were inoculated into another set of each of the bagged substrates. The substrates inoculated with *B. megaterium* and *A. niger* GIO were incubated at 28±2 ℃ for 5 and 10 days, respectively. After incubation, 40 ml of sterile distilled water was added to each bag, mixed and filtered. The filtrate was centrifuged at 1790 *g* for 15 min and the supernatant was regarded as the crude xylanase and used for further studies.

### Xylanase assay

The activity of xylanase was determined based on the reducing sugars released from beechwood xylan (Sigma-Aldrich, Germany) using the dinitrosalicylic acid (DNS) method [25]. Reaction medium was prepared by adding 0.5 ml of crude enzyme to 0.5 ml 1 % xylan in acetate buffer (pH 5.0, 0.5 M) prepared from analytical grades of sodium acetate and acetic acid. The mixture was incubated at 50 °C for 30 min and then the reaction was terminated by adding 1 ml of DNS reagent. The mixture was heated in boiling water for 10 min and the optical density was determined with a spectrophotometer (721G Visible Spectrophotometer, PR China) at 540 nm. The optical density of different concentrations of xylose was also determined and used to extrapolate the xylose sugar released. One unit of enzyme activity was defined as the amount of enzyme required to produce 1 μm xylose sugar min^−1^ in standard conditions.

### Effect of temperature on xylanase activity

Crude enzyme (0.5 ml) was added to 0.5 ml 1 % beechwood xylan (Sigma-Aldrich, Germany) in acetate buffer (pH 5.0, 0.5 M). The mixture was incubated at different temperatures (30, 40, 50, 60 °C) for 1 h at an interval of 15 min. The enzyme activities were determined according to the DNS method as described earlier for the xylanase assay.

### Effect of pH on xylanase activity

Crude enzyme (0.5 ml) was added to 0.5 ml 1 % beechwood xylan (Sigma-Aldrich, Germany) in different buffers of different pH (0.5 M acetate buffer: 4.0, 4.6, 5.0; 0.5 M phosphate buffer: 6.2, 6.8) and incubated at 50 °C for 30 min. The enzyme activities were determined according to the DNS method as described earlier for the xylanase assay.

### Effect of metal ions and surfactants on xylanase activity

The effects of different 5 mM metal ions (K^+^, Na^+^, Ca^2+^, Mg^2+^, Fe^2+^, Al^3+^) and 5 mM surfactants [ethylenediaminetetraacetic acid (EDTA), sodium dodecyl sulfate (SDS) and urea] on the activities of xylanase were determined by incubating crude xylanase with these additives at 50 °C for 30 min at pH 5.0. The enzyme activities were determined according to the DNS method as described earlier for the xylanase assay.

### Statistical analysis

The obtained experimental data were analysed using analysis of variance (ANOVA) to determine the means with SPSS version 23 and the level of significance was set at *P*≤0.05. GraphPad Prism 6.0.1 (GraphPad Software, Inc., USA) was used for graphical presentation.

## Results

### Screening for xylanase

**Fig. 1.**
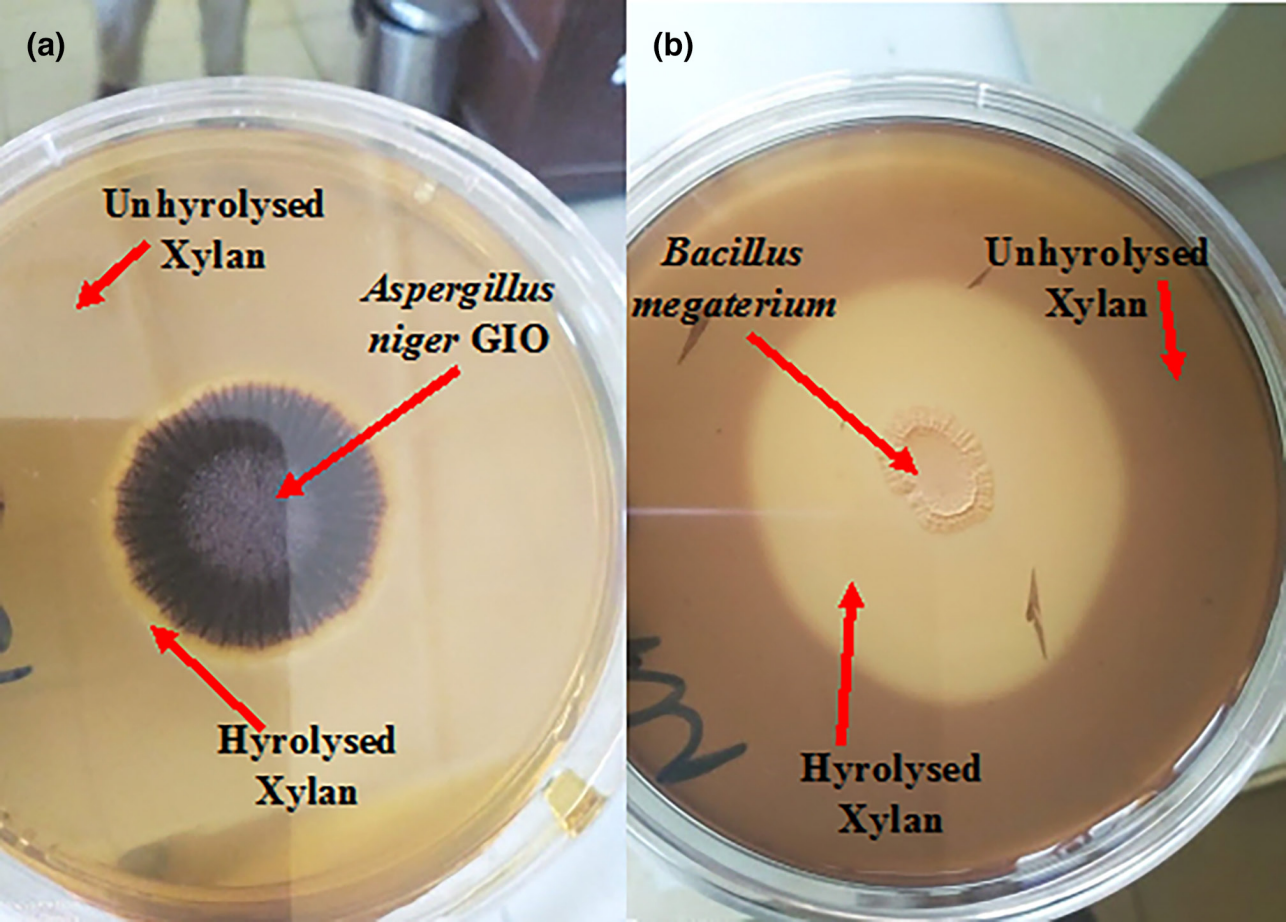
Xylanolytic activities of (**a**) *A. niger* GIO and (**b**) *B. megaterium* on xylan agar.

The ability of *A. niger* GIO and *B. megaterium* to hydrolyse xylan on media supplemented with xylan is shown in Fig. 1a, b. A clear zone around the point of inoculation against the dark colour of undegraded xylan indicates that *A. niger* GIO (Fig. 1a) and *B. megaterium* (Fig. 1b) produced xylanase.

### Production of xylanase

**Fig. 2.**
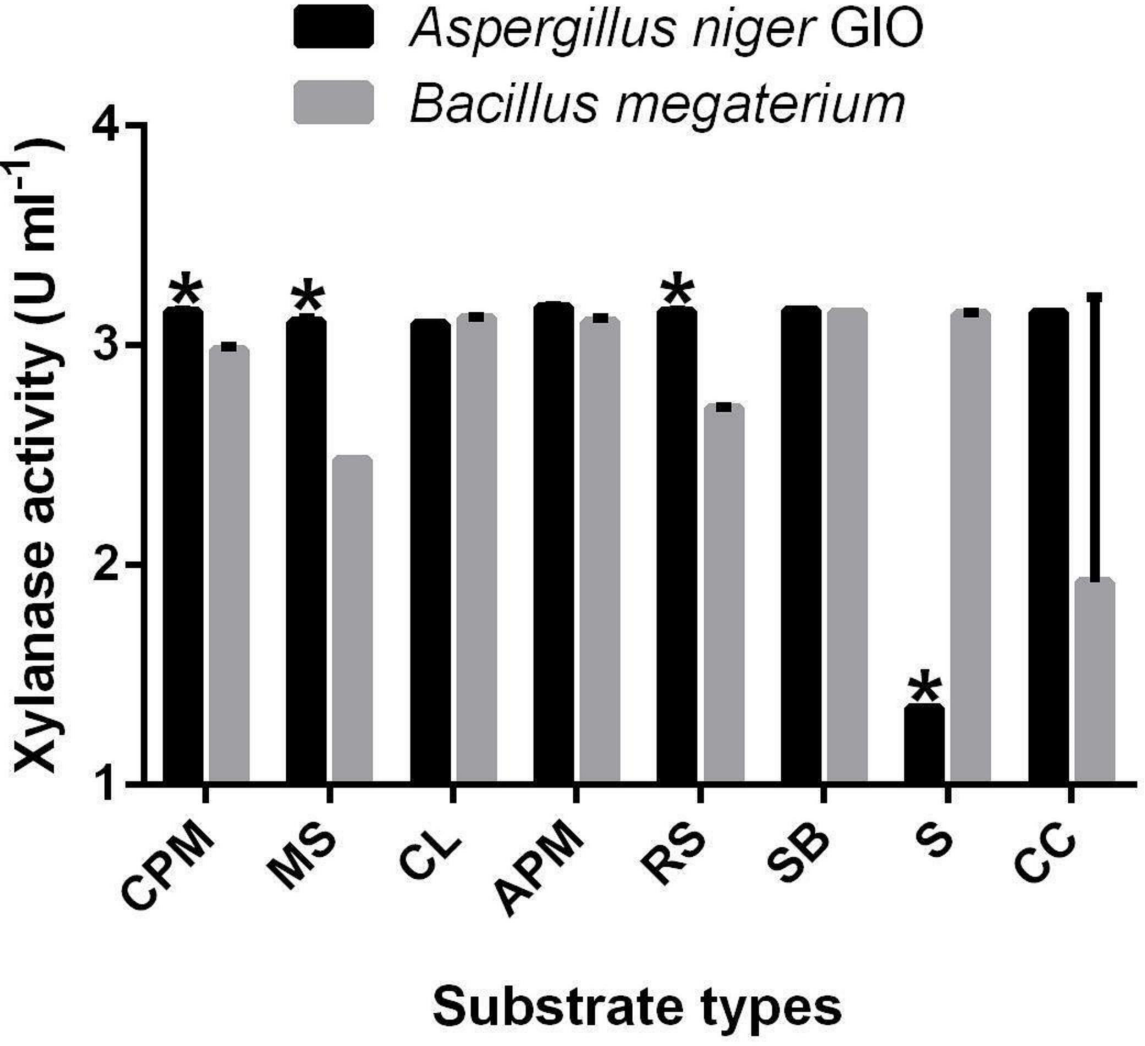
Solid-state fermentation of different agro-materials as substrates for xylanase production. CPM: Combined (biological and alkaline) pretreated maize straw. Data are the means of two replicates of xyanase activites. Error bar: Standard error of the mean. *Xylanase activity of *Aspergillus niger* GIO is significantly different when compared with *Bacillus megaterium* using same substrate at *P*<0.05. MS: Maize straw. CL: Conifer litters. APM: Alkaline-pretreated maize straw. RS: Rice straw. SB: Sugarcane baggase. S: Sawdust. CC: Corn cob.

All substrates served as positive solid substrate production components for xylanase production. Fig. 2 shows the activities of xylanase produced on different agro-materials by *A. niger* GIO and *B. megaterium *. The xylanase activities of *A. niger* GIO and *B. megaterium* were significantly different (*P*≤0.05) from each other with combined pretreated maize straw, maize straw, rice straw and sawdust as substrates. The activities of xylanase produced by *A. niger* GIO on different agro-wastes as substrate ranged from 1.35 U ml^−1^ (sawdust) to 3.18 U ml^−1^ (APM). The highest activity of xylanase (3.18 U ml^−1^) produced by *A. niger* GIO using APM as a substrate was significantly different (*P*≤0.05) from those produced using other substrates. The highest activity of xylanase (3.15 U ml^−1^) produced by *B. megaterium,* was recorded when sugarcane baggase was used as the substrate. This was followed by the 3.14 U ml^−1^ activity realized with the use of sawdust. However, the least activity (1.93 U ml^−1^) was obtained when corn cob was the substrate for production. The activity of xylanase produced using APM by *B. megaterium* (3.11 U ml^−1^) was not significantly different (*P*>0.05) from the highest activity of xylanase (3.15 U ml^−1^) produced by the same organism using sugarcane baggase as substrate. APM substrate was selected for further studies.

### Characterization of produced xylanase

#### Effect of temperature and incubation time

The effects of temperature and time on the activities of microbial xylanases are as shown in Table 1. The activities of xylanase produced by *A. niger* GIO and *B. megaterium* increased with an increase in temperature from 30 to 40 °C and decreased thereafter. At 30 °C, the activities of xylanase produced by *A. niger* GIO increased from 2.86 U ml^−1^ at 15 min to 3.31 U ml^−1^ at 30 min before a decrease in activities was recorded at 45 min. However, statistical analysis revealed that there was no significant difference (*P*>0.05) in activities recorded at 30, 45 and 60 min. The activities of xylanase produced by *B. megaterium* at 30 °C increased from 2.22 U ml^−1^ (15 min) to 3.13 U ml^−1^ (45 min), which then reduced to 2.88 U ml^−1^ after 60 min. The highest activities of xylanase produced by *A. niger* GIO (3.67 U ml^−1^) and *B. megaterium* (3.36 U ml^−1^) were recorded at a temperature of 40 °C at 30 and 45 min of incubation, respectively. At 40 °C, xylanase activities of *B. megaterium* at different incubation periods were not significantly different (*P*>0.05). A slight decrease in the activities of xylanase produced by *A. niger* GIO and *B. megaterium* were observed as the temperature increased to 50 °C, where the activities of xylanase produced by *A. niger* GIO and *B. megaterium* at different periods of incubation ranged from 3.28 to 3.41 U ml^−1^ and 3.01 to 3.21 U ml^−1^, respectively, indicating a respective 7.1 and 4.5% reduction when compared to that at 40 °C. Statistical analysis revealed that there was no significant difference (*P*>0.05) in the activities of xylanase produced by *A. niger* GIO at different incubation periods. Further decrease in activities of xylanase produced by both *A. niger* GIO and *B. megaterium* was observed as the temperature of incubation increased to 60 °C. Compared to the peak activity recorded at 40 °C, at 60 °C the highest activity of xylanase produced by *A. niger* GIO (3.26 U ml^−1^) was recorded at 45 min of incubation, while that of *B. megaterium* (3.24 U ml^−1^) was recorded at 60 min of incubation, resulting in a respective 11.2 and 3.6% reduction in enzyme activity. Hence the xylanase produced by both micro-organisms was thermotolerant and thermostable at the temperatures tested. There was no significant difference (*P*>0.05) in the activities of xylanase produced by *A. niger* GIO at different times of incubation at 60 °C.

**Table 1.** Effect of temperature on the activities of microbial xylanases (U ml^−1^) Mean values (±standard deviation) with different superscripts along the column are significantly different (*P*≤0.05).

Temp. (°C)	Time (min)	*A. niger* GIO	* B. megaterium *
30	15	2.86±0.062^a^	2.22±0.049^a^
	30	3.31±0.003^bcde^	3.05±0.029^bcde^
	45	3.18±0.130^bcde^	3.13±0.000^bcdef^
	60	3.19±0.214^bcde^	2.88±0.000^b^
			
40	15	3.38±0.001^def^	3.11±0.311^bcdef^
	30	3.67±0.143^g^	3.28±0.311^def^
	45	3.46±0.054^efg^	3.36±0.012^f^
	60	3.66±0.104^fg^	3.30±0.104^ef^
			
50	15	3.28±0.021^bcde^	3.01±0.007^bcde^
	30	3.37±0.071^de^	3.21±0.149^cdef^
	45	3.41±0.078^defg^	3.04±0.184^bcde^
	60	3.36±0.042^cde^	3.20±0.127^cdef^
			
60	15	3.05±0.065^ab^	2.99±0.019^bcd^
	30	3.16±0.115^bcd^	2.94±0.039^bc^
	45	3.26±0.097^bcde^	2.94±0.052^bc^
	60	3.07±0.311^abc^	3.24±0.259^cdef^

#### Effect of pH

**Fig. 3.**
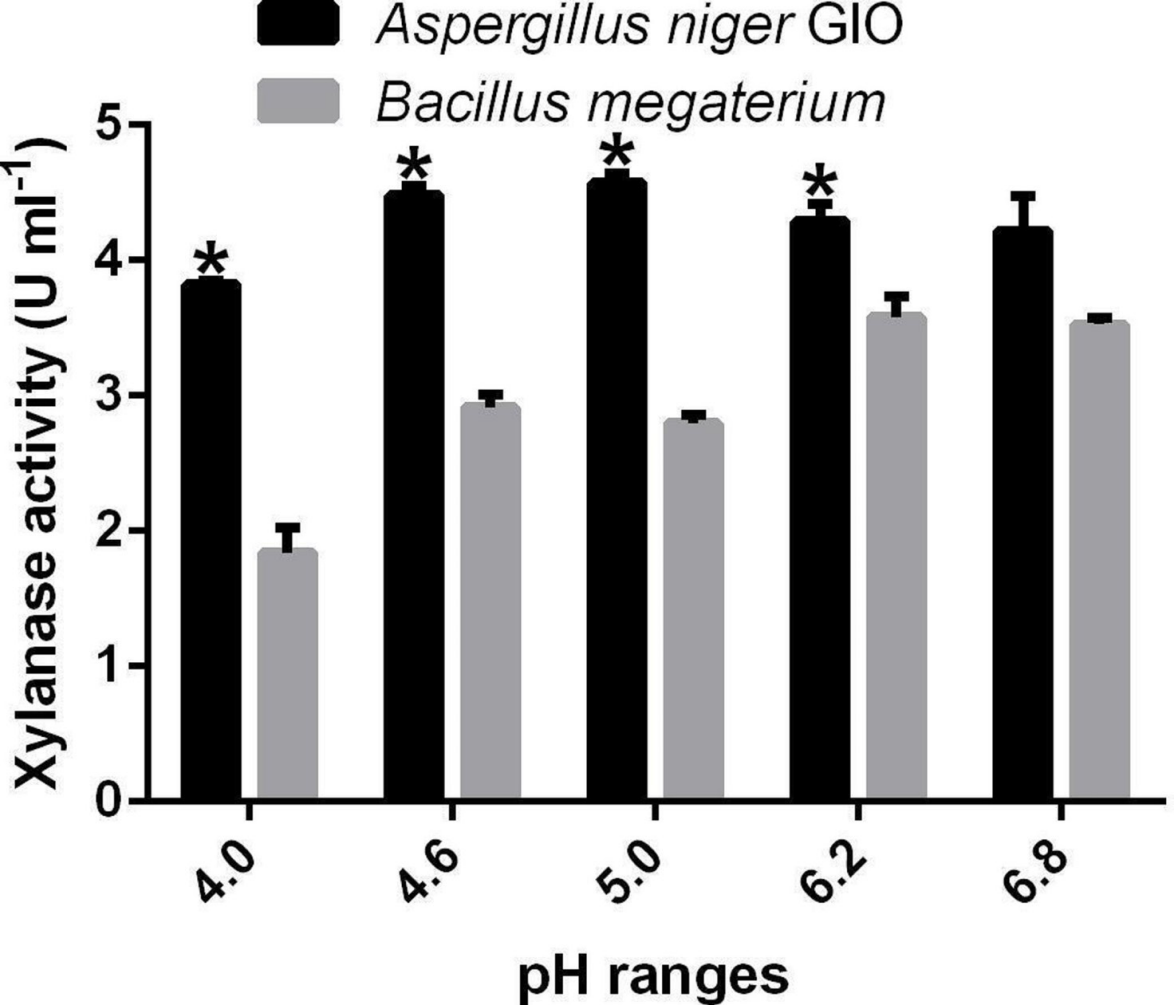
Effect of pH on the activities of microbial xylanases. Error bar: Standard error of the mean. *Xylanase activity of *Aspergillus niger* GIO is significantly different when compared with *Bacillus megaterium* at the same pH at *P*<0.05.

Fig. 3 shows the effect of pH on the activities of xylanase produced by *A. niger* GIO and *B. megaterium *. The activities of xylanase of *A. niger* GIO increased from 3.83 U ml^−1^ at pH 4.0 to 4.58 U ml^−1^ and thereafter decreased to 4.22 U ml^−1^ at pH 6.8. All activities of xylanase of *A. niger* GIO at all pH were not significantly different (*P*>0.05) from one another except the activity obtained at pH 4. Xylanase produced by *B. megaterium* had the highest activity (3.58 U ml^−1^) at pH 6.2. The activities of xylanase of *B. megaterium* recorded at pH 6.2 (3.58 U ml^−1^) and at pH 6.8 (3.53 U ml^−1^) were not significantly different (*P*>0.05).

#### Effect of metal ions and surfactants

**Fig. 4.**
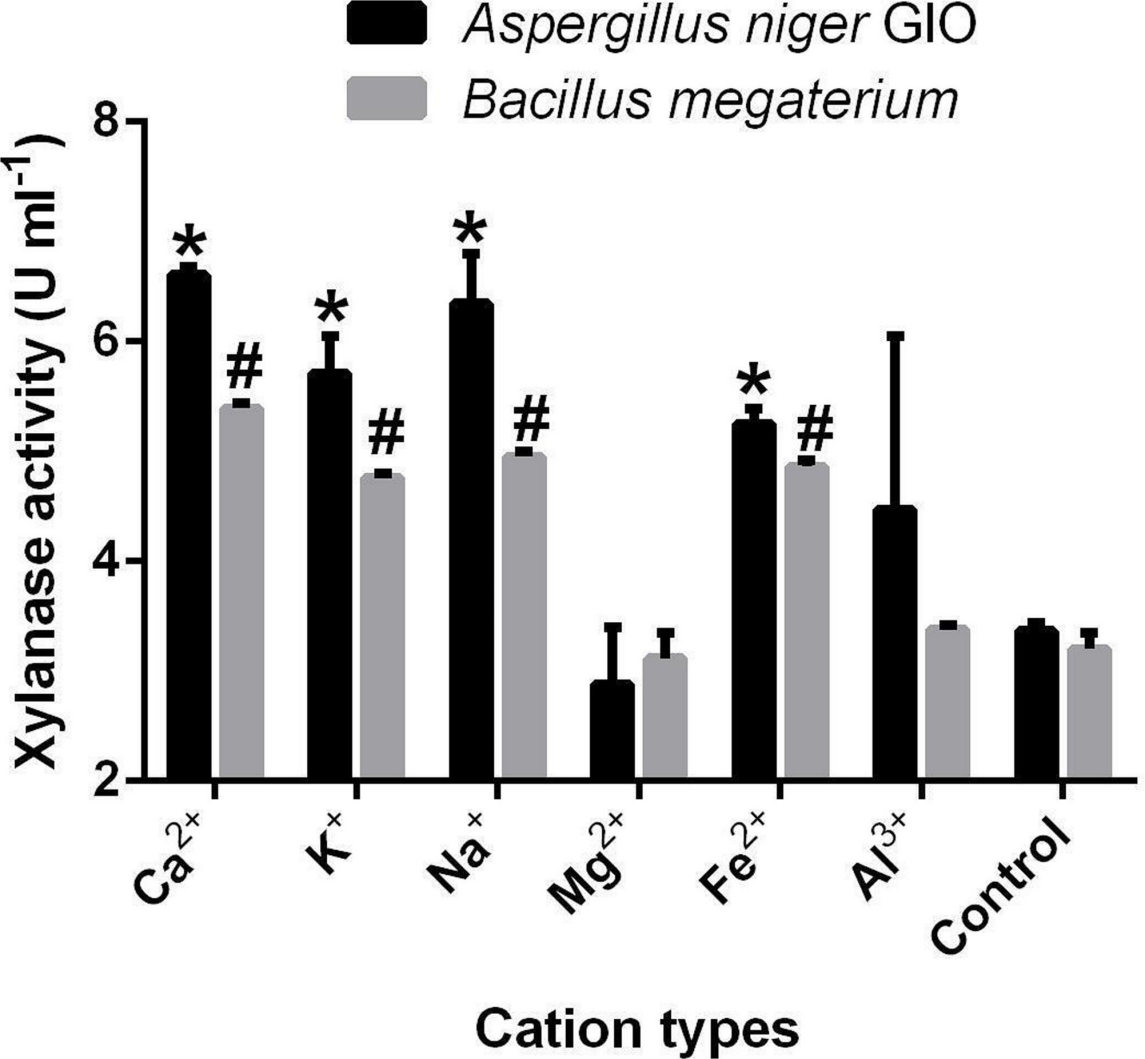
Effect of metal ions on the activities of microbial xylanases. Data are the means of two replicates of xylanase activities. *Xylanase activity of *Aspergillus niger* GOI is significantly different when compared with the control (*P*<0.05). #Xylanase activity of *Bacillus megaterium* is significantly different when compared with the control (P<0.05). Error bar: Standard error of the mean. Ca^2+^: Calcium ion; K^+^: Potassium ion; Na^+^: Sodium ion; Mg^2+^: Magnesium ion; Fe^2+^: Ferrous ion; Al^3+^: Aluminum ion.

Metal ions enhanced the activities of the xylanase produced by both *A. niger* GIO and *B. megaterium* (Fig. 4). With Ca^2+^, the highest activities of the *A. niger* GIO xylanase was 6.60 U ml^−1^ while that of *B. megaterium* was 5.40 U ml^−1^. These values were significantly different (*P*≤0.05) from their respective controls (without metal ion). Activities of xylanase of *A. niger* GIO and *B. megaterium* were also improved with iron II ion, sodium ion, and potassium ion. However, the least activities were observed with magnesium ions for both xylanases of *A. niger* GIO (2.88 U ml^−1^) and *B. megaterium* (3.12 U ml^−1^). When compared to the controls, the magnesium ion-influenced xylanase from *A. niger* GIO and *B. megaterium,* respectively had 85.5 and 97.2% xylanase activity. The activities of xylanase of *A. niger* GIO and *B. megaterium* were not significantly different from their respective control (without metal ions) when magnesium and aluminium ions were used.

The effect of surfactants on the activities of microbial xylanase is shown in Table 2. The highest activities of xylanase of *A. niger* GIO (6.13 U ml^−1^) and *B. megaterium* (6.90 U ml^−1^) were positively influenced by sodium dodecyl sulfate and were significantly different from their respective control (without surfactant). The values of activity recorded with urea by *A. niger* GIO (5.51 U ml^−1^) and *B. megaterium* (4.20 U ml^−1^) were significantly higher than their respective controls. There was no activity of microbial xylanase recorded when EDTA was used as a surfactant.

**Table 2.** Effect of surfactants on the activities of microbial xylanases (U ml^−1^) Mean values (±standard deviation) with different superscripts along the column are significantly different (*P*≤0.05).

Surfactant	*A. niger* GIO	* B. megaterium *
EDTA	0.00±0.000^a^	0.00±0.000^a^
SDS	6.13±0.075^d^	6.90±0.031^d^
Urea	5.51±0.047^c^	4.20±0.031^c^
Control	3.37±0.071^b^	3.21±0.149^b^

EDTA, ethylenediaminetetraacetic acid; SDS, sodium dodecyl sulfate.

## Discussion

*Aspergillus* and *Bacillus* species have been reported to produce a wide range of enzymes, especially lignocellulosic-degrading enzymes, which are involved in the breaking down of agricultural wastes [13, 16, 26]. *A. niger* GIO and *B. megaterium* used in this work hydrolysed xylan, with evidence of a clear zone on hyrolysed xylan media flooded with iodine against the dark colour of unhydrolysed xylan, as reported by Shakoori *et al*. [27].

Agro-wastes were used to produce xylanase in this study and the utilization of agro-wastes for the production of xylanase is a means of turning wastes into wealth. Many researchers have converted wastes to value-added products such as ethanol [28–32], with xylanase production from biomass residues as another example [10, 33–35]. Bastos *et al*. [11] used different agro-wastes (corn cob, barley bagasse, bacaba, rice husk, corn straw, pineapple crown, cassava husk and soybean husk) for the production of xylanase. Peach-palm waste was used for the production of xylanase by Carvalho *et al*. [22]. Zehra *et al*. [15] utilized banana peels for xylanase production. Utilization of wastes in the environment for the production of value-added products will create a safe environment and improve the economy.

The highest xylanase activities of *A. niger* GIO and *B. megaterium* were recorded at 40 °C in this study, which was similar to the work of Roy and Rowshanul [36], who reported the highest activities of xylanase being produced by a *Bacillus* species at 40 °C. Carvalho *et al*. [22] and Hernandez *et al*. [6] recorded the highest xylanase activity for their micro-organisms at 50 °C and 55 °C, respectively. The xylanase of *A. niger* GIO and *B. megaterium* in our work still retained 88.8 and 96.4% activity, respectively, at 60 °C when incubated for 45 and 15 min, respectively. This served as an indication of the thermostable properties of the xylanase produced. Generally, the optimal temperature for xylanase activity depends on the organisms producing it and xylanase activity is sensitive to temperature.

There was no significant difference in the xylanase activity of *A. niger* GIO from pH 4.6 to 6.8, which showed that the enzyme was relatively pH-stable and could be used effectively over an acidic to neutral pH range. Enzymes with relatively stable activities over pH ranges are important biotechnologically. The activities of xylanase are affected by pH because substrate bindings and catalysts depend on charge distributions of enzymes and substrates [37]. The optimal pH for the xylanase activities of *A. niger* GIO and *B. megaterium* were recorded at 5.0 and 6.2, respectively. Fungi have the ability to grow better than bacteria under an acidic environment and the activities of their enzymes are expected to be best at acidic ranges. It had been reported that acidic pH (3.0–5.5) favoured the activities of xylanase produced by fungi [10]. Bacteria grow better at a pH within the neutral region and their enzymes are expected to be active around this pH. Panthi *et al*. [37] reported an optimal pH of 6.0 for the xylanase activity of a *Bacillus* sp., while Hernandez *et al*. [6] recorded the highest xylanase activity for a bacterium at pH 6.5.

The addition of 5 mM potassium, sodium, calcium, iron (II) and aluminium metal ion improved the xylanase activities of *A. niger* GIO and *B. megaterium *, whereas magnesium ion repressed xylanase activities. Ferraz *et al*. [10] recorded an increase in xylanase activities when sodium, calcium and aluminium ions were added. The increase in xylanase activities observed with some metal ions could be a result of the ability of the metal ion to stimulate the active site of enzymes [12]. The highest activity was recorded when calcium ion was used as a metal ion, which was similar to the report of Hernandez *et al*. [6]. Calcium ions are required structurally to maintain the active site of xylanase, while its absence will have negative effects on the recognition of substrate by the active site [6]. The reduction of xylanase activities upon the addition of magnesium ion corroborated the findings of Hernandez *et al*. [6], who also reported a reduction in xylanase activities upon the addition of magnesium as a metal ion. The decrease in enzyme activities by some metal ions might be due to the formation of insoluble complexes when the metal ions and xylan are mixed [37].

The addition of EDTA to the microbial xylanase had a negative impact on the activities of microbial enzyme in this research, and no activity was recorded in the presence of EDTA. The inhibitory effect of EDTA indicated that xylanases of *A. niger* GIO and *B. megaterium* are metallo enzymes. Metallo enzymes are inhibited by EDTA and EDTA could act as a chelator, trapping metals that are required for proper enzyme folding [6, 13]. The xylanase activity of *Bacillus subtilis* JJBS250 was inhibited in the presence of EDTA [12]. Hernandez *et al*. [6] referred to EDTA as an organic acid inhibitor. Surfactants generally influence the function of proteins in cell signalling. Sodium dodecyl sulfate had a positive influence on the xylanase activities of the micro-organisms used in this work, but Gama *et al*. [19] and Sipriyadi *et al*. [20] reported that the addition of sodium dodecyl sulfate led to a decrease in the xylanase activities of different *Streptomyces* spp.

*B. megaterium* is a good producer of xylanase. Kareem *et al*. [38] used *B. subtilis *, *B. megaterium *, *Bacillus cereus* and *Escherichia coli* to produce xylanase and reported that *B. megaterium* was the best producer of xylanase among the different bacterial strains used in xylanase production. The xylanase activities of *A. niger* GIO (6.60 U ml^−1^) and *B. megaterium* (6.90 U ml^−1^) in this work were higher than the highest xylanase activity (3.6 U ml^−1^) recorded by Sipriyadi *et al*. [20], despite the different optimization steps the authors subjected the organism to.

## Conclusion

*A. niger* GIO and *B. megaterium* utilized an array of agro-wastes as substrates for the production of xylanase. The highest activity of the xylanase produced by *A. niger* GIO and *B. megaterium* was recorded at 40 °C, while more than 88 % activity was still realized at 60 °C. The xylanases were active over the different pH ranges (pH 4.0 to 6.8). Potassium, sodium, calcium, iron II and aluminium ions significantly improved the activities of the xylanase produced by *A. niger* GIO and *B. megaterium *. Sodium dodecyl sulfate supported the best xylanase activities from both micro-organisms. The utilization of agro-wastes, which would otherwise have been a source of pollution in the environment, for xylanase production resulted in the conversion of wastes into wealth.

## References

[1] Fasiku SA, Wakil SM (2021). Pretreatment of maize straw with *Pleurotus ostreatus* and *Lentinus squarrosulus* for bioethanol production using *Saccharomyces cerevisiae*. NRMJ.

[2] Fasiku SA, Wakil SM (2022). Screening of factors responsible for conversion of maize straw into bioethanol. J microb biotech food sci.

[3] Glekas PD, Kalantzi S, Dalios A, Hatzinikolaou DG, Mamma D (2022). Biochemical and thermodynamic studies on a novel thermotolerant GH10 xylanase from *Bacillus safensis*. Biomolecules.

[4] Kucharska K, Rybarczyk P, Hołowacz I, Łukajtis R, Glinka M (2018). Pretreatment of lignocellulosic materials as substrates for fermentation processes. Molecules.

[5] Chukwuma OB, Rafatullah M, Tajarudin HA, Ismail N (2020). Lignocellulolytic enzymes in biotechnological and industrial processes: a review. Sustainability.

[6] Balderas Hernández VE, Salas-Montantes CJ, Barba-De la Rosa AP, De Leon-Rodriguez A (2021). Autodisplay of an endo-1,4-β-xylanase from *Clostridium cellulovorans* in *Escherichia coli* for xylans degradation. Enzyme Microb Technol.

[7] Ullah S, Irfan M, Sajjad W, Rana QUA, Hasan F (2019). Production of an alkali-stable xylanase from *Bacillus pumilus* K22 and its application in tomato juice clarification. Food Biotechnol.

[8] Bhardwaj N, Kumar B, Verma P (2019). A detailed overview of xylanases: an emerging biomolecule for current and future prospective. Bioresour Bioprocess.

[9] Tundo S, Paccanaro MC, Bigini V, Savatin DV, Faoro F (2022). The *Fusarium graminearum* FGSG_03624 xylanase enhances plant immunity and increases resistance against bacterial and fungal pathogens. IJMS.

[10] de Almeida Antunes Ferraz JL, Oliveira Souza L, Gustavo de Araújo Fernandes A, Luiz Ferreira Oliveira M, de Oliveira JR (2020). Optimization of the solid-state fermentation conditions and characterization of xylanase produced by *Penicillium roqueforti* ATCC 10110 using yellow mombin residue (*Spondias mombin* L.). Chem Eng Commun.

[11] Alves de Bastos LT, Dias Batista R, Martins Lima AC, Leandro dos Santos I, Coutinho de Paula-Elias F (2021). Production of xylanase by *Fusarium oxysporum* using agro-industrial waste. Sci Plena.

[12] Alokika S, Kumar V, Singh B (2023). Biochemical characteristics of a novel ethanol-tolerant xylanase from *Bacillus subtilis* subsp. subtilis JJBS250 and its applicability in saccharification of rice straw. Biomass Conv Bioref.

[13] Köstekci S, Aygan A, Comlekcioglu N, Sarıtürk S (2022). Alkaline cellulase free xylanase from *Bacillus* sp. ASX42: properties, purification and its effect on seed germination.. J microb biotech food sci.

[14] Cunha L, Martarello R, de Souza PM, de Freitas MM, Barros KVG (2018). Optimization of xylanase production from *Aspergillus foetidus* in soybean residue. Enzyme Res.

[15] Zehra M, Syed MN, Sohail M (2020). Banana peels: a promising substrate for the coproduction of pectinase and xylanase from *Aspergillus fumigatus* MS16. Pol J Microbiol.

[16] Tuyen DT, Cuong NT, le Thanh NS, Thao NT, Hoang LT (2021). Cloning, expression, and characterization of xylanase G2 from *Aspergillus oryzae* VTCC-F187 in *Aspergillus niger* VTCC-F017. Biomed Res Int.

[17] Ullah SF, Souza AA, de Freitas SM, Noronha EF (2022). Characterisation of biomass degrading xylanolytic enzymes of *Penicillium chrysogenum* produced using sugarcane bagasse. Process Biochem.

[18] Cruz-Davila J, Perez JV, Castillo DS del, Diez N (2022). *Fusarium graminearum* as a producer of xylanases with low cellulases when grown on wheat bran. Biotechnol Rep.

[19] Gama AR, Brito-Cunha CCQ, Campos ITN, de Souza GRL, Carneiro LC (2020). *Streptomyces thermocerradoensis* I3 secretes a novel bifunctional xylanase/endoglucanase under solid-state fermentation. Biotechnol Prog.

[20] Sipriyadi S, Wahyudi AT, Suhartono MT, Meryandini A (2020). Optimization of xylanase production by *Streptomyces costaricanus* 45I-3 using various substrates through submerged fermentation. Microbiol indones.

[21] Isil S, Nilufer A (2005). Investigation of factors affecting xylanase activity from *Trichoderma harzianum* 1073 D3. Braz arch biol technol.

[22] Carvalho EA, Nunes LV, Goes LM, Silva EG, Franco M (2018). Peach-palm (bactris gaspaes kunth.) waste as substrate for xylanase production by trichomderma stromaticum AM7. Chem Eng Commun.

[23] Sneath PH, Mair NS, Sharpe ME, Holt JG (1986). Bergey’s Manual of Systematic Bacteriology.

[24] Pointing SB (1999). Qualitative methods for the determination of lignocellulolytic enzyme production by tropical fungi. Fungal Divers.

[25] Miller GL (1959). Use of dinitrosalicylic acid reagent for determination of reducing sugar. Anal Chem.

[26] Fasiku SA, Ogunsola OF, Fakunle A, Olanbiwoninu AA (2020). Isolation of bacteria with potential of producing extracellular enzymes (Amylase, Cellulase and Protease) from soil samples. JAMB.

[27] Shakoori FR, Shokat M, Saleem F, Riaz T (2015). Screening, optimization and characterization of xylanase by locally isolated bacteria. Punjab Univ J Zool.

[28] Wakil SM, Fasiku SA, Adelabu AB, Onilude AA (2013). Production of bioethanol from spontaneous fermentation of palm oil mill effluent (POME). Researcher.

[29] Japar AS, Takrif MS, Jahim JM, Kadhum AA (2017). The effect of glucose addition in acetone butanol-ethanol fermentation from palm oil mill effluent by *Clotridium acetobutylicum* NCIMB 619. MJAS.

[30] Mohammad S, Baidurah S, Kamimura N, Matsuda S, Bakar NASA (2021). Fermentation of palm oil mill effluent in the presence of *Lysinibacillus* sp. LC 556247 to produce alternative biomass fuel. Sustainability.

[31] Prasertsan P, Leamdum C, Chantong S, Mamimin C, Kongjan P (2021). Enhanced biogas production by co-digestion of crude glycerol and ethanol with palm oil mill effluent and microbial community analysis. Biomass Bioenerg.

[32] Wakil SM, Adelabu AB, Fasiku SA, Onilude AA (2013). Production of bioethanol from palm oil mill effluent using starter cultures. N Y Sci J.

[33] Azzouz Z, Bettache A, Djinni I, Boucherba N, Benallaoua S (2022). Biotechnological production and statistical optimization of fungal xylanase by bioconversion of the lignocellulosic biomass residues in solid-state fermentation. Biomass Conv Bioref.

[34] Tian M, Wai A, Guha TK, Hausner G, Yuan Q (2018). Production of endoglucanase and xylanase using food waste by solid-state fermentation. Waste Biomass Valor.

[35] Marques GL, Silva TP, Lessa OA, Brito AR, Reis NS (2019). Production of xylanase and endoglucanase by solid-state fermentation of Jackfruit residue. Rev Mex Ing Quim.

[36] Roy N, Rowshanul HM (2009). Isolation and characterization of xylanase producing strain of *Bacillus cereus* from soil. Iran J Microbiol.

[37] Panthi S, Choi YS, Choi YH, Kim M, Yoo JC (2016). Biochemical and thermodynamic characterization of a novel, low molecular weight xylanase from bacillus methylotrophicus CSB40 isolated from traditional Korean food. Appl Biochem Biotechnol.

[38] Rafid AWAK, Imrana K, Muhammad AB, Qureshi AS, Ayyaz A (2014). Xylanase production using fruit waste as cost effective carbon source from thermo-tolerant *Bacillus megaterium*. Afr J Microbiol Res.

